# Analysis of the Thickness of Ligamentum Flavum and Its Relationship With Degenerative Disc Changes at L3-4, L4-5, and L5-S1 Levels in Patients Undergoing Magnetic Resonance Imaging (MRI) of the Lumbosacral Spine

**DOI:** 10.7759/cureus.74233

**Published:** 2024-11-22

**Authors:** Gaurav Kumar, Anil Kumar Sakalecha, Jagannathan Krishnan, Mahima Kale R, Neelam Katre

**Affiliations:** 1 Radiodiagnosis, Sri Devaraj Urs Medical College, Kolar, IND

**Keywords:** degenerative disc disease, l3-l4, l4-l5, l5-s1 levels, ligamentum flavum hypertrophy, low back pain, lumbar spinal canal stenosis

## Abstract

Background

Low back pain (LBP) is the leading cause of disability among working-age adults, with its prevalence increasing with age and peaking in the 45-54 age group. It is common practice for clinicians to conduct advanced imaging procedures, such as computed tomography (CT) and magnetic resonance imaging (MRI) when a patient presents with LBP. The objective of this study was to measure and analyze the width of the ligamentum flavum (LF) on each side and the extent of degeneration of the disc at the L3-4, L4-5, and L5-S1 levels.

Methods

The current investigation was a cross-sectional descriptive-analytical effort carried out in the radiodiagnosis department of Sri Devaraj Urs Medical College, Tamaka, Kolar, from July 2024 to September 2024. The study's inclusion criteria were participants in age groups ranging from 20 to 60 years who were referred for an MRI of the lumbosacral spine and had a prior history of LBP.

Results

Out of 60 subjects, 26 (43.3%) individuals were females, and 34 (56.7%) individuals were males. Out of the maximum number of patients, 23 (38.3%) were within the age range of 31-40 years. The medial and lateral aspects of the LF measurements showed an increase with age, with lateral values being greater than the medial measurements (p = 0.001). The mean thickness of the ligament and the mean height of the disc showed significant variations at all levels. Statistically significant differences were seen in the thickness of the LF of male patients (4.82, 0.62 mm) compared to female patients (4.89, 0.72 mm). A statistically significant positive Pearson association was observed between the thickening of the LF and Pfirrmann grading of degenerative disc disease (DDD).

Conclusion

The LF thickness is significantly associated with disc degeneration, especially at the L4-L5 level, contributing to spinal canal stenosis, which is critical for the management of LBP.

## Introduction

Low back pain (LBP) is one of the leading causes of disability among working-age adults; LBP is also a prevalent complaint seen in basic healthcare clinics [1]. LBP is the leading cause of disability worldwide, with its prevalence increasing with age and peaking in the 45-54 age group [2]. In high-income countries, LBP leads to significant clinical and direct and indirect economic burdens. The prevalence of chronic LBP in sub-Saharan Africa ranges from 18.1% to 28.2% in the general population, highlighting its global reach [3,4].

It is common practice for clinicians to conduct advanced imaging procedures, such as computed tomography (CT) and magnetic resonance imaging (MRI), when a patient presents with LBP [1]. One of the ligaments that reinforce and maintain the joints between vertebrae is the ligamentum flavum (LF). LF attaches superiorly to the upper lamina and inferiorly to the lower lamina [2]. A possible explanation for the constriction of the canal of the spinal cord and physical compression of the spinal nerve roots or the cauda equina is the thickening of the LF [3]. An increase in the thickness of the lumbar LF can lead to lumbar spinal canal stenosis (LSCS) and root compression pain, even in the absence of disc protrusion [1,4]. 

The relationship between LF hypertrophy and pain severity can be quantified using the visual analog scale (VAS), which shows a positive correlation with LF thickening [5]. Degenerative changes in the LF, often due to mechanical stress, are significant contributors to LBP and are associated with the degeneration of intervertebral discs [6]. The relationship between LF hypertrophy and degenerative disc disease (DDD) is complex, involving various factors such as age, sex, and specific lumbar levels [7]. LF thickening and its associated with disc degeneration is critical in understanding the pathophysiology of LBP and LSCS. This association is crucial for diagnosing and managing LBP effectively [8]. LF thickening is often associated with disc degeneration, particularly at the L4-L5 level, where it is more pronounced than at the L5-S1 level. This thickening is linked to increased mechanical stress and age-related degenerative processes, contributing to LBP [9]. A study found no direct correlation between LF thickness and disc height or degree of degeneration, suggesting that LF hypertrophy might not be a direct consequence of disc degeneration but rather a parallel degenerative process [10,11].

Insufficient knowledge exists regarding the relationship between LF hypertrophy and the degree of disc degeneration based on the spinal levels in patients experiencing LBP. Thus, the objective of this work was to measure and analyze the width of ligament flavum on each side and the extent of degeneration of the disc at the L3-4, L4-5, and L5-S1 levels.

## Materials and methods

The current investigation was a cross-sectional descriptive-analytical effort carried out in the radiodiagnosis department of Sri Devaraj Urs Medical College, Tamaka, Kolar, India. This study was submitted to and approved by the institutional ethical committee with EC no. SDUAHER/KLR/R&D/CEC/S/PG/51/2024-25. The study included a cohort of 60 individuals who were diagnosed with LBP and were referred for an MRI of the lumbosacral spine. The study's inclusion criteria were participants in age groups ranging from 20 to 60 years who were referred for an MRI of the lumbosacral spine and had a prior history of LBP. Exclusion criteria included the medical history of bone metastases, congenital abnormalities, scoliosis, discitis, osteomyelitis, traumatic spondylolysis, spondylolisthesis, ossification, or fracture. Furthermore, the study excluded patients who had claustrophobia, ferromagnetic spinal implants, pacemakers, and aneurysm clips. MRI scans were performed on all patients using the Siemens® MAGNETOM Avanto, a 1.5 Tesla, 18-channel MRI machine, in both sagittal and axial planes. The lumbosacral spine was analyzed using standard protocol MRI sequences, including sagittal T1- and T2-weighted sequences as well as axial T1- and T2-weighted sections. Each case is assessed by the evaluator to determine the extent of degeneration and thickness of the LF at the L3-4, L4-5, and L5-S1 levels.

**Figure 1:**
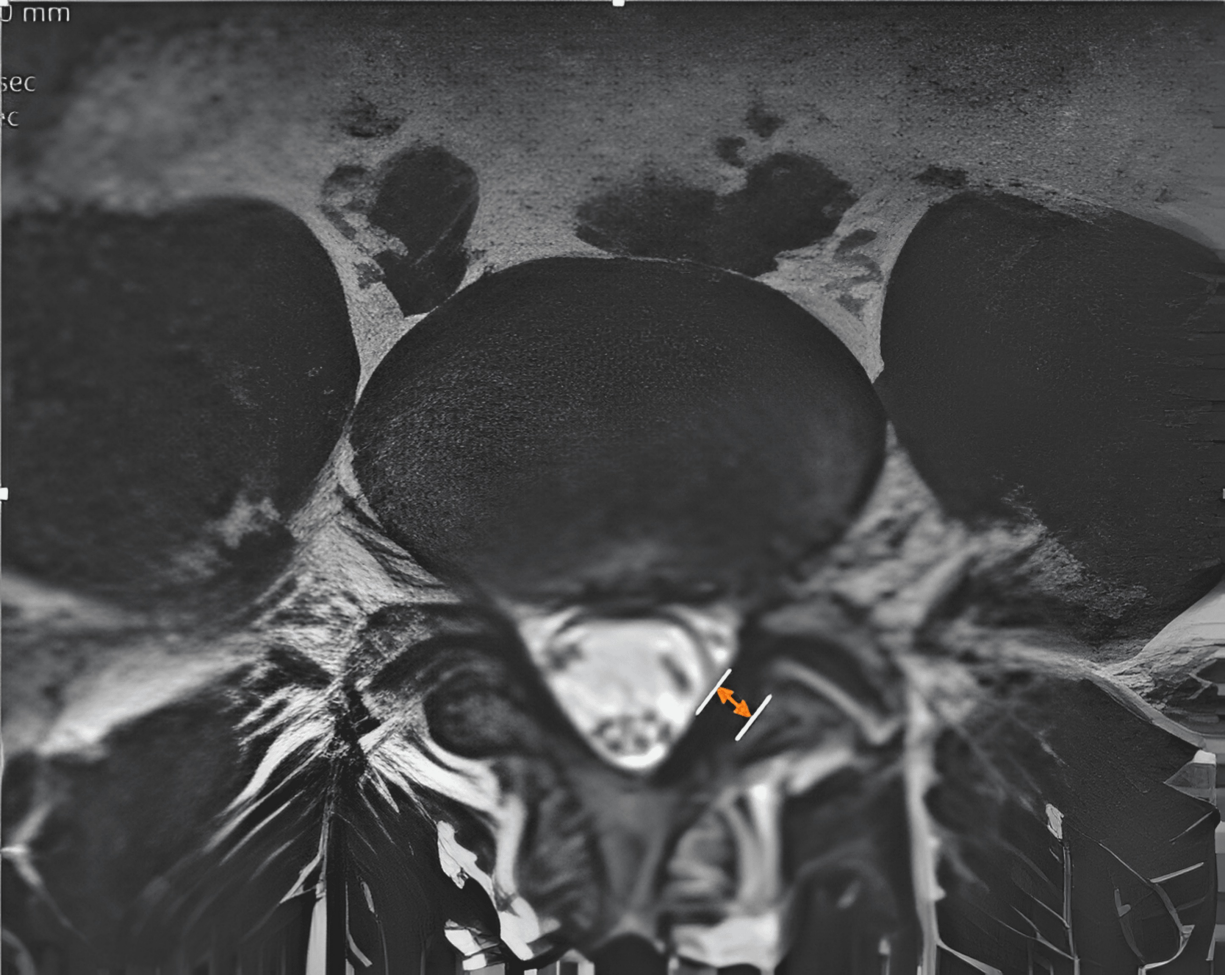
Measurement of the ligamentum flavum thickness on axial T2-weighted MRI image at the level of the facet joint MRI: magnetic resonance imaging Source: original image

The LF thickness was quantified in millimeters (mm) using a digital caliper at a precision of 0.1 mm on the T1-weighted axial slices that were orientated lateral to the spinal canal's plane and perpendicular to the layers at the facet joint level on both sides. Furthermore, the mean width of the LF was computed. If the thickness exhibited bilateral asymmetry, the measurements of the thickest section were employed. Figure 1 demonstrates the method of measuring the LF thickness.

An investigation conducted by Park et al. [5] classified a thickness over 4 mm as thickened LF. The assessment of degeneration of the disc was carried out using the method of Pfirrmann et al. [6], which considered the dissimilarity in disc structure, the differentiation between the core and the annulus, and the height of the disc. The disc degeneration was then graded from I to V.

Statistical data was entered into MS Excel (Microsoft Corporation, Redmond, Washington, United States) and subsequently analyzed using IBM SPSS Statistics for Windows, Version 22 (Released 2013; IBM Corp., Armonk, New York, United States). In this study, categorical data was expressed using frequencies and proportions. An analysis of significance for qualitative data was conducted using either the chi-square test or Fischer's exact test (for 2 x 2 tables only). Continuous data was expressed using measurements of mean and standard deviation. The independent t-test and Mann-Whitney U test were employed as appropriate tests of significance to determine the difference in means between two quantitative and qualitative variables, respectively. Measures of Pearson correlation and Spearman's correlation were used to determine the association between two quantitative and qualitative variables, respectively. A p-value of less than 0.05 was regarded as statistically significant.

## Results

A total of 60 patients who met the specified criteria were included in the trial over the designated period. Of the total, 26 (43.3%) individuals were females, and 34 (56.7%) individuals were males. The mean age of the patients considered in the research was 33 years. Out of the total number of patients, 23 (38.3%) were within the age range of 31-40 years, followed by 41-50 age group (25%) and 51-60 age group (23.3%), and the least encountered were in the age group of 21-30 years (Table 1).

**Table 1:** Age and gender demographic distribution in the study sample. Percentage is calculated with respect to the total number of subjects (total subjects: 60)

Gender	N (%)	Age (years)	N (%)
Males	34 (56.7%)	21-30	8 (13.3%)
Females	26 (43.3%)	31-40	23 (38.3%)
		41-50	15 (25%)
		51-60	14 (23.3%)

The L3-L4 LF thickness values fit well within the expected range for healthy individuals, and the minor differences between the right and left sides (RT LF being slightly thicker than LT LF) were common and not statistically significant. The thickness of the LF at the L4-L5 level was measured to be 5.4 ± 0.66 mm on the right side and 5.2 ± 0.62 mm on the left side. The thickness of the LF was assessed to be 5.1 ± 0.64 mm on the right side and 4.9 ± 0.61 mm on the left side at the L5-S1 level. As indicated in Table 2, the thickness of LF was found to be greater on the right side than on the left side at both intervertebral disc levels. The thickness of the LF exhibited a statistically significant rise with age. This statistical tendency was validated when the age group was analyzed at the spinal level. A comparable pattern was observed as the thickness of the LF increased with the older age group (p < 0.001). 

**Table 2:** Measurement of thickness of the ligamentum flavum at different intervertebral disc levels on both side RT: right; LT: left; LF: ligamentum flavum

Spinal level	Side	Mean (mm)	SD	Min (mm)	Max (mm)
L3-L4	RT LF	4.8	0.6	3	6.1
	LT LF	4.6	0.58	3.1	6
L4-L5	RT LF	5.4	0.66	3.7	6.7
	LT LF	5.2	0.62	3.8	6.6
L5-S1	RT LF	5.1	0.64	3.3	6.5
	LT LF	4.9	0.61	3.6	6.3

In this study, the mean thickness of the ligament and the mean height of the disc showed significant variations based on all the levels (p < 0.001). The pairwise comparisons in Table 3 indicated that the ligaments at L4-L5 had a significantly higher average thickness compared to ligaments at L5-S1 (p < 0.001). Similarly, the L3-L4 LF thickness had a mean of 4.90 mm, which was slightly less than that of L4-L5, with a standard deviation of 0.70 mm, indicating moderate variability. The p-value was significant at <0.001. The L3-L4 disc height showed a mean of 10.50 mm, smaller than the levels below, with a standard deviation of 2.00 mm, indicating variability among individuals. The p-value was significant at 0.0012. 

**Table 3:** Thickness of ligamentum flavum and intervertebral disc height of each level and the results of comparison between the variables LF: ligamentum flavum *Statistically significant

Parameters	Level	Mean (mm)	Median	Min (mm)	Max (mm)	SD	p-value
LF thickness	L3-L4	4.9	4.85	3.5	6.3	0.7	<0.001^*^
	L4-L5	5.13	5.20	3.68	6.71	0.77	
	L5-S1	4.79	4.75	3.71	6.38	0.64	
Disc Height	L3-L4	10.5	10.6	6.8	14.0	2.0	<0.001^*^
	L4-L5	11.38	11.45	7.15	15.98	2.18	
	L5-S1	12.27	12.3	7.23	12.58	2.43	

Nevertheless, the disc located at L5/S1 differs in height from the disc at the L4-L5 level. A one-way repeated measure analysis of variance (ANOVA) was performed to compare the thickness of the LF and the height of the discs at each level (p < 0.0012). An increase in the thickness of the LF was seen when the disc height dropped. The study revealed that males had greater disc height than females at the intervertebral disc level (Table 4).

**Table 4:** Intervertebral disc height and thickness of the ligamentum flavum according to gender, results of comparison between the sexes (student t-test) LF: ligamentum flavum

Parameter and levels	Gender	N	Mean	SD	p-value
Disc height at L3-L4	Male	34	10.65	2.1	0.045
	Female	26	10.43	2.05	
Disc height at L4-L5	Male	34	11.56	2.17	0.005
	Female	26	11.14	2.08	
Disc height at L5-S1	Male	34	12.34	2.15	0.041
	Female	26	11.87	2.71	
Avg. LF thickness at L3-L4	Male	34	4.95	0.65	0.035
	Female	26	4.75	0.6	
Avg. LF thickness at L4-L5	Male	34	5.14	0.63	0.034
	Female	26	5.25	0.79	
Avg. LF thickness at L5-S1	Male	34	4.82	0.62	0.024
	Female	26	4.89	0.72	

However, the thickness of the LF was seen to be greater in females (5.25 ± 0.79 mm) than that in males (5.14 ± 0.63 mm) at the L4-L5 level. Statistically significant differences were seen in the thickness of the LF of male patients (4.82 ± 0.62 mm) compared to female patients (4.89 ± 0.72 mm) at the L5-S1 level. 

A positive association between disc degeneration and LF thickness is seen in Table 5, indicating that the grade of disc degeneration increases with the thickening of the LF. Furthermore, the height of the disc exhibited a negative association with age, meaning that as the patient's age increases, the height of the disc decreases.

**Table 5:** Result of correlation of variables for each levels LF: ligamentum flavum *Statistically significant

Levels	Variables	N	Correlation	p-value
L3-L4	Age versus disc height only	60	-0.55	<0.001*
	Age versus LF thickness	60	0.44	
	Disc height versus LF thickness	60	-0.62	
	Pfirrmann versus LF thickness	60	0.71	
	Age versus Pfirrmann grading	60	0.63	
L4-L5	Age versus disc height only	60	-0.71	<0.001*
	Age versus LF thickness	60	0.66	
	Disc height versus LF thickness	60	-0.68	
	Pfirrmann versus LF thickness	60	0.82	
	Age versus Pfirrmann grading	60	0.58	
L5-S1	Age versus disc height only	60	-0.71	<0.001*
	Age versus LF thickness	60	0.41	
	Disc height versus LF thickness	60	-0.51	
	Pfirrmann versus LF thickness	60	0.68	
	Age versus Pfirrmann grading	60	0.56	

A statistically significant positive Pearson association was observed between the thickness of the LF and Pfirrmann grading. The link between LF thickness and disc degeneration was shown to be more significant at the L3-L4 and L4-L5 levels compared to the L5-S1 level (r = 0.82, p < 0.001 at L4-L5 the level; r = 0.68, p < 0.001 at the L5-S1 level).

## Discussion

The development of LSCS is significantly influenced by posterior spinal disorders, particularly the hypertrophy of the LF [7]. In addition to physical factors, degenerative impairments are the primary etiology of LBP, a prevalent cause of disability among persons of working age. Alterations in LF thickness are associated with degenerative developments in the lumbar spine and increase the likelihood of experiencing clinical symptoms [3].

The objective of this work was to analyze and determine the thickness of LF on both sides and the extent of disc degeneration at the L3-L4, L4-L5, and L5-S1 levels. Significantly, the bulk of individuals experiencing LBP belonged to the younger age bracket, with a nearly equal distribution of males and females. This phenomenon may be attributed to the significant reliance of the younger generation on physical labor. Observations by Shoukat et al. [8] indicate that young adults suffering from LBP frequently experience disc degeneration at lower lumbar levels, mainly at L4-L5 and L5-S1. There are no notable gender-based differences in spinal involvement, but different underlying variables contribute to this phenomenon. While Karki et al. and Mannion et al. conducted studies that showed a significant percentage of males seeking healthcare services for LBP, the present study shows similar results [9-11].

The current study indicates that the thickness of the LF varies with age, and notable variations in LF thickness were observed at the L4-L5 and L5-S1 levels as individuals age. The results of this investigation were consistent with the research conducted by Altinkaya et al. [12], which demonstrated a favorable association between age and the thickness of the LF at the L4-L5 level. The study conducted by Wang et al. [13] revealed a favorable correlation between age and LF thickness at the L4-L5 level in individuals with LSCS. The current investigation bears resemblance to the study conducted by Sakamaki et al. [14], which demonstrated a statistically significant rise in the thickness of the LF at the L4-L5 and L5-S1 levels as individuals age. Furthermore, the study indicated that a thickness exceeding 4 mm was observed in the age group of 20-40 years, which was attributed to mechanical stress in the younger population.

The results of this investigation are particularly relevant given previous studies that have shown the highest thickness of the LF at the L4-L5 level. The average thickness in these studies varied from 3.5 to 5.5 mm, which is comparable to the thickness seen in our current study. Khasawneh et al. [15] observed that the thickness of the LF at the L4-L5 level does not significantly grow with age. However, there are greater increases at the L4-5 and L3-4 levels compared to the L2-L3 and L5-S1 levels. MRI revealed that the LF had its maximum thickness at the L4-L5 level, measuring 3.71 ± 0.29 mm. The MRI study conducted by Kolte et al. [2] revealed that the thickness of the LF was greatest at the L4-L5 level. A study conducted by Mattar et al. [11] revealed that the thickness of the LF was highest at the L4-L5 level in patients suffering from persistent LBP. A further investigation conducted by Ramani et al. [16] and Spurling et al. [17] determined that the thickness of the LF was much greater. The variation in the thickness of the LF among the several investigations could be attributed to the sample group, which encompassed both younger individuals (under 20 years) and older respondents (over 60 years).

The present investigation revealed a right-sided more LF thickening as compared to the left at both the L4-L5 and L5-S1 levels, which is consistent with the findings reported by Abbas et al. [18] and Sudeep et al. [1]. The scientists have described this asymmetry to the inherent rotation of the right lumber region, which results in increased thickening of the right LF.

Contrary to that, the study conducted by Safak et al. [19] found that the left frontal fat is thicker on the left side because of individual preferences for that side. A study conducted by Hansson et al. [20] examined the thickness of the LF using MRI before and after an external axial load. The study indicated that buckling of the LF may result in central canal stenosis. A negative association was seen between the height of the disc and the thickness of the LF at all three levels in our investigation. The outcome of this investigation was consistent with the findings of Sudeep et al. [1] and Mattar et al. [11], who examined the relationship between disc height and LF thickness. Their results showed that as disc degeneration increased, disc height dropped. Although the degeneration of the disc is a gradual and continuous process, the bulging of the LF may develop due to reduced elasticity of the LF. This bulging then leads to tissue injury, inflammation, scarring, and ultimately fibrosis as the patient ages.

Limitations

First of all, a variety of conditions, such as spondylolisthesis and facet osteoarthritis, can cause the spinal canal to narrow beyond the thickness of the LF. These more variables should be incorporated into the analysis in order to have a deeper understanding of the pathophysiology of canal narrowing. Our study group was also limited in number and did not include a control group. Consequently, it was not possible to assess the relationship between LF thickness and LSCS symptoms within the parameters of our investigation. Our study didn’t include subjects beyond 60 years. Future research with participants over 60 might provide insight into the degenerative cascade of spondylosis, which includes LF hypertrophy.

## Conclusions

Previous research in the area of spine biomechanics has suggested a clear correlation between age and LF thickness. Our investigation has verified that there is a progressive enlargement of the LF as age progresses at the lumbar level, occurring up to the L4/L5 level with maximum effect at the L4-L5 level. The thickness of the LF was found to be positively correlated with the degree of degeneration of the disc and the subsequent decrease in vertebral height.
